# The Recycling of Substandard Rocket Fuel N,N-Dimethylhydrazine via the Involvement of Its Hydrazones Derived from Glyoxal, Acrolein, Metacrolein, Crotonaldehyde, and Formaldehyde in Organic Synthesis

**DOI:** 10.3390/ijms242417196

**Published:** 2023-12-06

**Authors:** Elizaveta Ivanova, Margarita Osipova, Tatyana Vasilieva, Alexey Eremkin, Svetlana Markova, Ekaterina Zazhivihina, Svetlana Smirnova, Yurii Mitrasov, Oleg Nasakin

**Affiliations:** 1 Organic and Pharmaceutical Chemistry Department, Ulyanov Chuvash State University, Moskovsky Prospect, 15, 428015 Cheboksary, Russia; lizachimic@mail.ru (E.I.); margolev1966@icloud.com (M.O.); tava52@mail.ru (T.V.); eremkin80@mail.ru (A.E.); konxmark@mail.ru (S.M.); eiz2020@mail.ru (E.Z.); svet0110lana@mail.ru (S.S.); 2 Organic and Pharmaceutical Chemistry Department, Yakovlev Chuvash State Pedagogical University, K. Marx Street, 38, 428000 Cheboksary, Russia; mitrasov_un@mail.ru

**Keywords:** asymmetric dimethylhydrazine, methylendimethylhydrazone, glyoxal dimethylhydrazone, acrolein dimethylhydrazone, crotonal dimethylhydrazone

## Abstract

“Heptil” (unsymmetrical dimethylhydrazine—UDMH) is extensively employed worldwide as a propellant for rocket engines. However, UDMH constantly loses its properties as a result of its continuous and uncontrolled absorption of moisture, which cannot be rectified. This situation threatens its long-term usability. UDMH is an exceedingly toxic compound (Hazard Class 1), which complicates its transportation and disposal. Incineration is currently the only method used for its disposal, but this process generates oxidation by-products that are even more toxic than the original UDMH. A more benign approach involves its immediate reaction with a formalin solution to form 1,1–dimethyl-2-methylene hydrazone (MDH), which is significantly less toxic by an order of magnitude. MDH can then be polymerized under acidic conditions, and the resulting product can be burned, yielding substantial amounts of nitrogen oxides. This review seeks to shift the focus of MDH from incineration towards its application in the synthesis of relatively non-toxic and readily available analogs of various pharmaceutical substances. We aim to bring the attention of the international chemical community to the distinctive properties of MDH, as well as other hydrazones (such as glyoxal, acrolein, crotonal, and meta-crolyl), wherein each structural fragment can initiate unique transformations that have potential applications in molecular design, pharmaceutical research, and medicinal chemistry.

## 1. Introduction

At normal temperature and pressure, asymmetric dimethylhydrazine (UDMH, 1,1-Dimethylhydrazine, heptil) is a hygroscopic liquid that appears colorless or slightly yellowish. It has the chemical formula (CH_3_)_2_N_2_H_2_, a relative molecular weight of 60.08, and a density of 785 kg/m^3^. UDMH has a boiling point of +63 °C and a crystallization temperature of −57 °C.

UDMH exhibits high solubility in water, alcohols, ammonia, amines, and organic solvents while being insoluble in hydrocarbons. It is a potent reducing agent [1]. When burned, UDMH produces highly toxic volatile nitro compounds [2] and releases a significant amount of energy. Due to these properties, it is widely utilized as a fuel in rocket technology. It is employed in domestic intercontinental ballistic missiles such as R36M2 “Voevoda”, as well as launch vehicles like “Cosmos”, “Cyclone”, and “Proton”. Additionally, UDMH is used in propulsion systems of manned spacecraft, automatic satellites, orbital and interplanetary stations, as well as reusable spacecraft [3,4].

However, UDMH exhibits marked toxicity [5], teratogenicity, and the capacity to absorb atmospheric moisture, leading to a loss of fuel characteristics [2,3,4]. Rectification methods are unable to counteract the water absorption (up to 2% annually). Consequently, aqueous heptil must be transported over long distances while implementing special precautions to processing facilities and then returned. Any incidents during UDMH transport constitute environmental disasters, resulting in a significant increase in the cost of “restored” UDMH. Therefore, it is more economical and safer to dispose of large quantities (thousands of tons!) at designated storage locations. The current approach involves an immediate exothermic reaction with formalin [6], yielding 1,1-dimethyl-2-methylene hydrazone (MDH) with reduced toxicity on an order of magnitude. Subsequently, MDH is polymerized under acidic conditions, followed by incineration [4]. However, even this relatively safe method imposes substantial harm on the environment due to the emission of significant amounts of nitrogen oxides, considering the disposal of thousands of tons of UDMH. From an ecological and economic perspective, locally processing the UDMH presents itself as the optimal and sole viable solution to the existing problem.

This review aims to show the relatively few possibilities and alternative ways of UDMH treatment [4] resulting in less toxic hydrazones (formaldehyde, glyoxal, acrolein crotonal, metacrolein) and their chemical transformations into the building blocks of UDMH-based bioactive organic compounds, using the literature from around the world up to 2022.

Currently, a notable instance of utilizing unsymmetrical dimethylhydrazine (UDMH) in the field of medicine is exemplified by the compound meldonium, which serves as an active constituent within the pharmaceutical preparation known as “Mildronate” [7]. This particular substance has gained significant recognition due to its association with doping scandals in the realm of sports. Owing to the inherent toxicity and challenges associated with handling UDMH within laboratory settings (where even the mere detection of UDMH odor surpasses sixfold the maximum permissible concentration), we propose the adoption of non-toxic derivatives of UDMH, namely, dimethyldrazones, such as glyoxal, acrolein, metacrolein, and formaldehyde, for employment both within chemical laboratories and industrial contexts.

## 2. Glyoxal Monodimethylhydrazone

Mono(dimethylhydrazon) glyoxal (**DMHG**, monohydrazon) is a compound of significant scientific interest in the field of organic chemistry due to its potential as a versatile synthon for the synthesis of multifunctional and biologically active structures. **DMHG** can be readily synthesized by combining unsymmetrical dimethylhydrazine (UDMH) and glyoxal in an aqueous solution under magnetic stirring, followed by extraction of the desired product using methylene chloride and subsequent vacuum distillation [8]. **DMHG** is characterized as a slightly yellowish liquid with a boiling point of 90 °C at 16 Torr [8].

### 2.1. Stereoselectivity of DMHG

The stereoselectivity of **DMHG**, which is recognized as one of its significant advantages, holds great importance in the field of medicine. This is because spatial isomers of the same compound exhibit distinct properties and varying degrees of harmful effects on pathogens and the human body. Utilizing **DMHG** as a starting material, optically pure alpha-aminoaldehydes have been successfully synthesized [9,10].

This achievement is particularly challenging due to the racemic nature of alpha-aminoaldehydes, which complicates their separation via chromatographic methods [11]. Consequently, **DMHG** has served as a valuable precursor for diverse compounds such as interleukin-converting enzymes (an enzyme responsible for converting interleukin, a mediator of the immune system, into a protein), calpains (a calcium-dependent cysteine protease that plays a role in protein degradation and cellular mobility) [12], amino alcohol intermediates, peptide analogs [13], organometallic complexes [9,14,15] utilized in the fabrication of thin optical films, and magnesium–copper alloys [15], among other important derivatives.

Likewise, the compound based on dimethylhexahydroxyflavylium (**DMHG**) [16] was employed to synthesize optically pure polymetinnitrile dyes, which hold potential as photosensitizers for antimicrobial photodynamic therapy.

**Figure 1:**
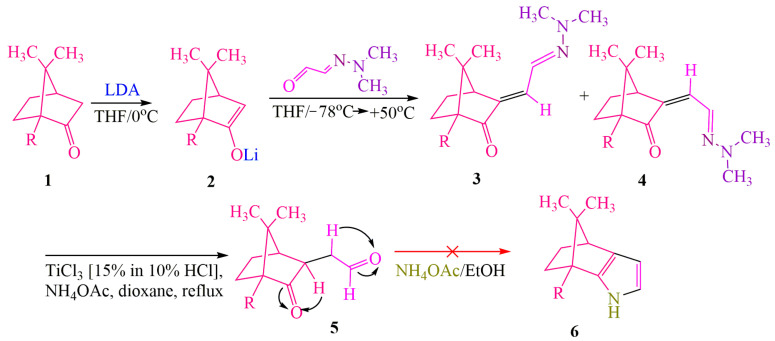
Annealing of camphor with pyrrole heterocycle (method 1). The red cross indicates that ammonium acetate is an incorrect reagent for the cyclization of compound 5 into pyrrole 6.

**Figure 2:**
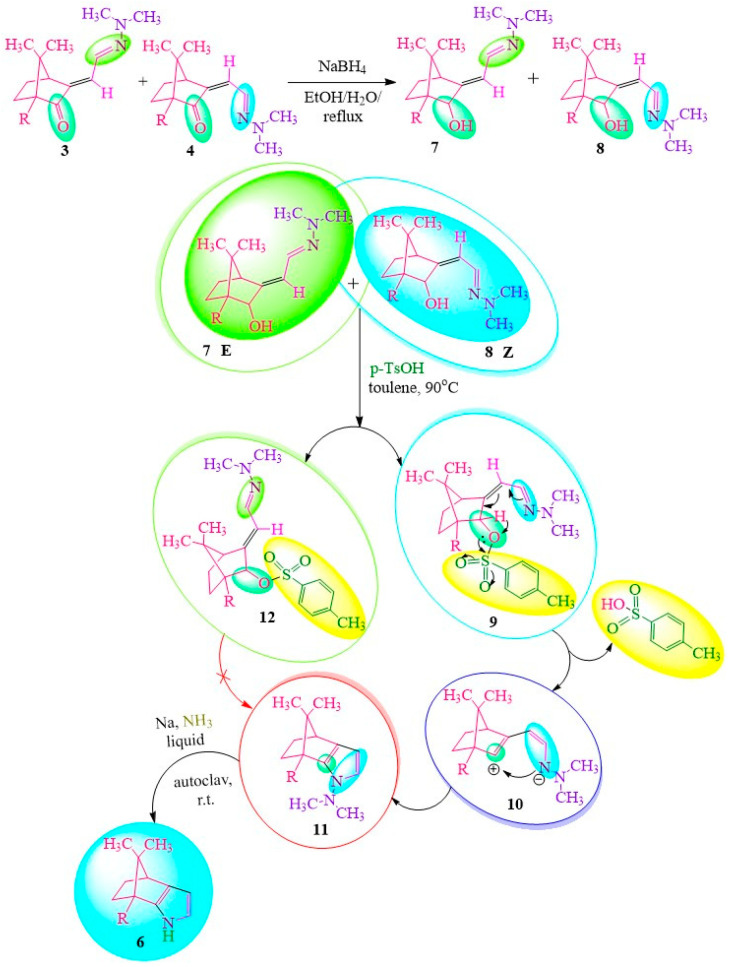
Annealing of camphor with pyrrole heterocycle (method 2).

Furthermore, the publication [17] explores the directed synthesis of a chiral auxiliary reagent based on **DMHG**. The aim is to obtain an optically pure, biologically active derivative of camphor. Figure 1 and Figure 2 in the publication outline the synthetic pathways. In their work, the authors performed condensation of **DMHG** with camphor **1** [17] (Figure 1). To facilitate this reaction, lithium diisopropylamide (**LDA**) was utilized for several reasons:

**LDA**, being a strong base, stabilizes the lithium enolate formed during the reaction. The α-position in camphor experiences steric hindrance. The reaction was carried out at the maximum temperature acceptable for the process involving **LDA** in tetrahydrofuran (THF), which was 0 °C [18]. It has been observed that the interaction between **DMHG** and enolate **2** is temperature-dependent. At −78 °C, the equilibrium shifts towards lithium alcoholates, while at +50 °C, it favors the formation of the desired isomers **3(E)** and **4(Z)**. The racemic mixture of isomers 3/4 could be reduced to 1,4-dicarbonyl compound **5** using titanium chloride, eliminating the need for isomer separation. However, attempts to cyclize **5** with ammonium acetate resulted in the decomposition of the original compound (Figure 1).

In search of an alternative cyclization method (Figure 2), the authors of [17] selectively reduced the keto group to hydroxyl using a mixture of 3/4 sodium borohydride. This facilitated the elimination of p-toluenesulfonic acid (tosylic acid) as an easily detachable leaving group [19], subsequently leading to the closure of the pyrrole ring. The results demonstrated that only one isomer, specifically the Z-isomer, participated in the cyclization process, resulting in compound **4**. The N-N bond cleavage of the pyrrole ring (compound **11**) was accomplished by reacting it with sodium in liquid ammonia under stirring conditions in an autoclave at room temperature. This reaction pathway ultimately yielded the desired product, compound **6** (Figure 2). 

### 2.2. DMHG in the Directed Synthesis of Biologically Active Analogues of Natural Compounds and Potential Drugs

**Figure 3:**
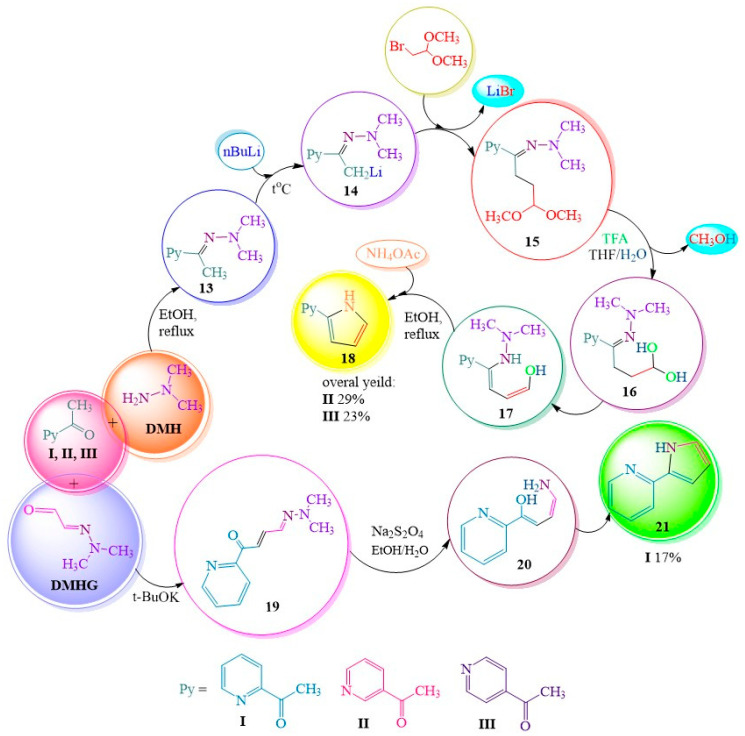
Preparation of pyrrol-2yl-pyridines by methods A (Knorr synthesis) and B.

**DMHG** is also promising in the creation of bioactive heterocycles (pyrroles, pyrazoles, isoxazoles) that contribute to many drugs [20,21,22,23,24,25,26,27,28,29,30,31,32,33,34,35,36] and alkaloids [37,38,39]. The authors of the publication [40] have also developed a strategy for cyclizing UDMH and its glyoxal derivative **DMHG** into biologically active pyrrole-2-ylpyridines (Figure 3). Among them, α-pyrrolylpyridine inhibits pyrrole-4-hydroxylase [40,41], which affects biosynthesis and collagen stability [41], while β-pyrrolylpyridine exhibits neuroprotective activity [42].

Thus, to obtain a pyrrole ring, the authors of [40] utilized intramolecular condensation employing the Knorr method. Initially, dimethylhydrazone **13** was subjected to metallization to yield compound **14**, which was subsequently converted into acetal **15**. Subsequently, hydrolysis of the acetal group in the presence of trifluoroacetic acid (TFA) took place. The elimination of water from compound **17** was followed by its intramolecular cyclization, resulting in the formation of the desired compound **18**. This method proved suitable for synthesizing pyrrole-pyridines **II** and **III**. However, in the case of pyrrole-pyridine **I**, which acts as a propyl-hydroxylase-I inhibitor, complications arose during the alkylation stage, leading to a reduction in yield. Consequently, the authors were motivated to explore an alternative pathway. A method was devised for the synthesis of α-analogues by condensing 2-acetylpyridine **I** with **DMHG** in the presence of potassium tert-butylate [43]. The latter acts as a potent base, effectively activating a “critical” terminal methyl group on ketone **I**. The resulting condensation product **19** was subsequently cyclized into pyrrole **21** with concurrent cleavage of the N-N bond [40], employing a safer alternative reagent, sodium dithionite, in comparison to the previously described method involving flammable sodium in an ammonia solution [17]. The authors of [40] reported successful and rapid preparation of compound **19**. However, during the subsequent step, a portion of the desired product **21** was lost due to its high volatility, resulting in a modest yield of only 17% (Figure 3).

Additionally, **DMHG** and its derivatives [44,45] have been extensively utilized in the synthesis of multicomponent alkaloids [37,38,39]. Among these, Isostemopholin [37] possesses insecticidal properties [46]. Furthermore, marine alkaloids such as Benz[c][2,7]naphthyridine, Amphimedine, Cystoditines, and Pyrido[4,3,2-mn]acridone [38] exhibit diverse and significant biological activities, including calcium ion release, antiviral effects, antimicrobial properties, and cytotoxicity against mouse leukemia cells (L1210) [38]. Moreover, inhibitory activity has been observed against lymphoma (assessed using the L1210 cell line, IC_50_ = 9.7 µg/mL), carcinoma (evaluated on the KB cell line, IC_50_ > 10 µg/mL), and cholinesterase [39].

Publication [38] describes the **DMHG**-based synthesis of the marine alkaloid Pyrido[4,3,2-mn]acridone. A monohydrazone fragment is introduced into the pyridine ring using **LDA** (lithium diisopropylamide). The synthesis was conducted in tetrahydrofuran (THF) at −70 °C to maintain kinetic control and prevent the decomposition of **LDA**, as it deprotonates the target product rapidly [18]. Lithiation of pyridine **22** finally occurred at the β-position instead of γ-position. The authors of [38] explain it refering to the rearrangement of pyrazoles (“dancerearrangement”) in publication [47].

**Figure 4:**
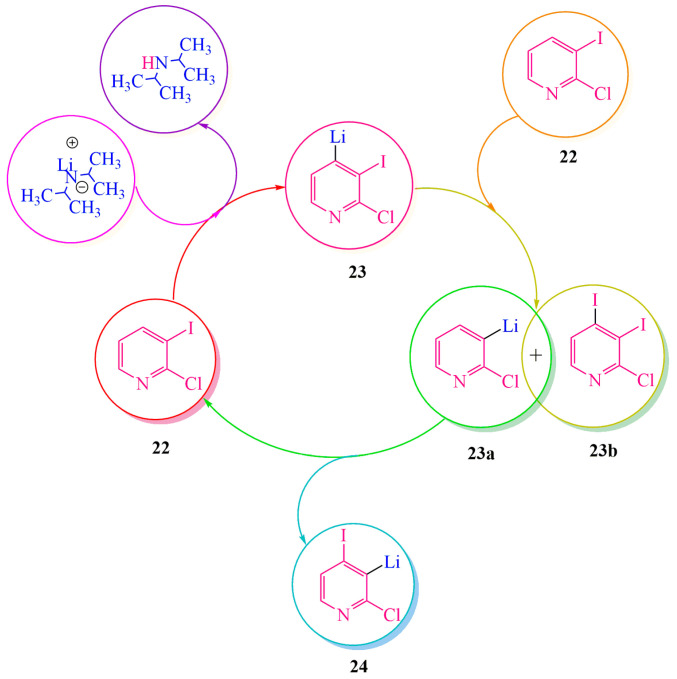
Interaction **22** with **LDA.**

The transition from intermediate **23** to **24** proceeded through multiple stages (Figure 4). Due to its high basicity, lithium derivative **23** interacted with the starting reagent 24 through an ion exchange mechanism, leading to the formation of 3-lithium-2-chloropyridine **23a** and 3,4-diiodo-2-chloropyridine **23b**. The interaction between these compounds resulted in the rearranged product 24 and the simultaneous regeneration of the initial compound **22**, which then reentered the cyclic process until complete conversion to the intermediate compound **24**. The lithiated derivatives directly interacted with the iodide ion, which exhibited higher reactivity compared to the chloride ion (Figure 4).

**Figure 5:**
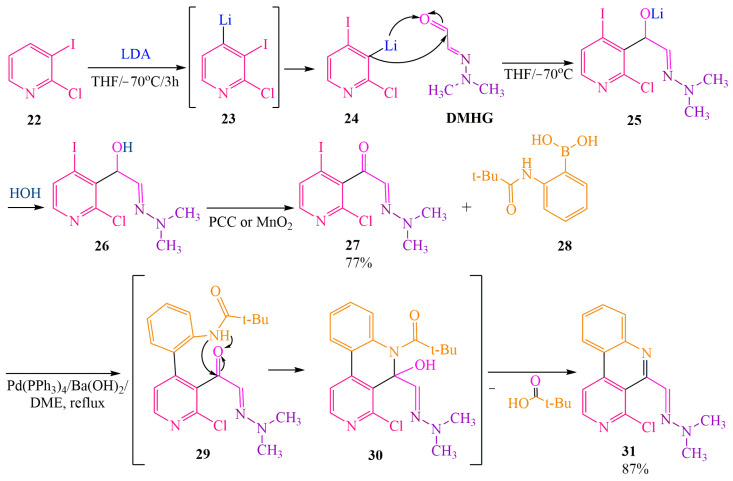
Preparation of acetylpyridine is followed by cross-coupling.

According to the authors of [38], pyridine lithiation (Figure 5) occurred at the γ-position, followed by the transformation of intermediate **23** into a more stable form, **24**. 

Compound **24** and DMHG, upon interaction, yielded alcohol **26**. This product was subsequently oxidized to acetylpyridine **27** using either manganese dioxide or pyridinium chlorochromate (PCC), resulting in a 77% yield [38] (Figure 5). Following this, the authors of [38] employed a cross-coupling reaction. In contrast to the conventional Suzuki conditions involving potassium carbonate and diglim (bis-2-methoxyethyl ether) [48], the authors utilized barium hydroxide and dimethoxyethyl (DME) as a more basic system. This modification allowed for an increased yield of the desired product **31** (87%) obtained through the cross-coupling of acetylpyridine **27** with boronic acid **28**, followed by intramolecular cyclization of amide **29** and elimination of tert-butyl carboxylic acid **30** (Figure 5).

**Figure 6:**
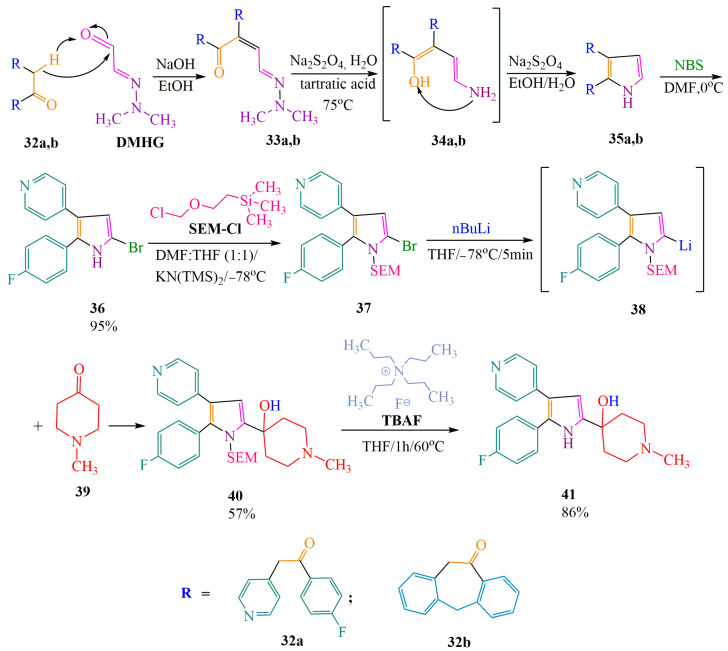
Intramolecular cyclization into a pyrrole ring and further chemical transformations of compound **35a**.

Attempts have been made to synthesize inhibitors of p38 MAP kinases (mitogen-activated protein kinases) based on **DMHG** [49,50,51], aiming to reduce the production of pro-inflammatory cytokines, which contribute to tissue destruction in diseases such as rheumatoid arthritis, an inflammatory joint disease. In one method [49] (Figure 6), the authors propose a **DMHG**-based condensation in an alkaline medium, alcohol solution, without the use of organometallic reagents. This approach is chosen because the original aldehydes **32a,b** do not hinder enolization due to the arrangement of atoms. The authors of [49,50] performed cyclization of hydrazones **33a,b** followed by N-N bond cleavage using a sodium dithionite aqueous alcohol solution, similar to the method described above [40] (Figure 6).

The resulting heterocycle **35a**, when interacting with bromosuccinimide (**NBS**), underwent further chemical transformations (Figure 6) into 2-bromopyrrole **36**. The halogenation was followed by lithiation providing 2-lithiumpyrrole **38**. Its condensation with N-methylpiperidone **39** led to the product **40** with a yield of 57%. However, the organometallic agent BuLi has well-defined basic properties and, therefore, interacts with an acidic heteroatom. In order to avoid adverse reactions, the authors of [49] introduced SEM-protection ((2-(chlorometoxy)ethyl)trimethylsilane) followed by pyrrole **37** lithiation and its condensation with N-methylpiperidine **39**. Tretbutylammonium fluoride (**TBAF**) [52] when allowing mild conditions of the reaction and providing a good yield of the target product **41**, was selected in order to remove SEM-protection of compound **40**.

In the case of 4-[5-(4-fluorophenyl)-4-pyridine-4-yl-1H-pyrrol-2-yl]-1-methyl-piperidine-4-ol **41** [49], biological activity was confirmed with an IC_50_ value of 0.13 µM. However, 1H-8-oxa-1-aza-dibenzo[e,h]azulen1H-dibenzo[2,3:6,7]azepino[4,5-b]pyrrol [51,53] was not detected (IC_50_ > 10 µmol dm^−3^), indicating a lack of inhibitory activity. Table S1 in the Supplementary Materials presents data collected by the authors of the article [49] on the inhibitory activity of 4-[5-(4-fluorophenyl)-4-pyridine-4-yl-1H-pyrrol-2-yl]-1-methyl-piperidine-4-ol derivatives, including **DMHG**, along with other heterocycles of similar structures. According to [49], the five compounds exhibit significant inhibition of p38α kinase, with IC_50_ values in the range of 10^−6^ M. Among these compounds, the pyrrole synthesized based on **DMHG** demonstrates the second highest inhibitory activity after imidazole (see Table S1 in the Supplementary Materials).

**Figure 7:**
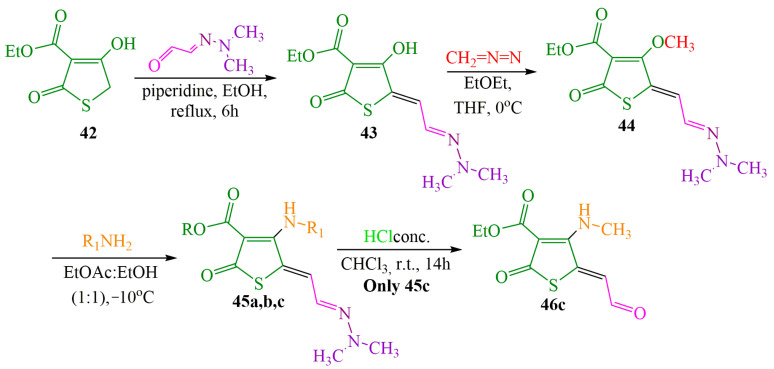
Synthesis of thiobazidalin.

The investigated compound (**DMHG**) was utilized in the synthesis of lesser-known thiobazidalin antibiotic derivatives [54] (Figure 7). In the initial step, the authors of publication [54] performed the condensation of thiolactone **42**, derived from tetronic acid, with **DMHG** in the presence of piperidine. Subsequently, hydrazone **43** reacted with diazomethane in cooled THF to prevent diazomethane ignition. Consequently, methoxy groups **44a**,**c** were converted into amino groups **45a,c** (refer to substituents in Table 1) by adding ammonia or methylamine to an EtOAc:hexane (1:1) solution at −10 °C. The desired compound **46c** was obtained by hydrolyzing hydrazone **45c** in a concentrated hydrochloric acid medium. Acid **45b** was obtained through the hydrolysis of ester **45a** in a dry acetonitrile solution under a nitrogen atmosphere. The authors likely chose **DMHG** as a reagent for two reasons: it possesses a protective group, and the electron saturation induced by the carbonyl nitrogen in hydrazone **45a,b,c** enhances the reactivity of the aldehyde group (Figure 7).

The reagents and conditions are presented in Table 1.

The authors of the publication [54] tested the compounds’ activity against various bacteria, such as Bacillus subtilis (“Hay bacillus”, involved in microbiocenoses of soil and human and mammalian intestines and found in water and in the air) and Bacillus brevis (bacteria found in water, air, soil, and decomposing organisms), and fungi, such as Mucor miehei (a type of fungus commercially used to produce renin (“rennet enzyme”) for milk production), *Paecilomyces varioti* (mold formed in rotting wood, soil and causing a number of infectious diseases in humans, such as ostiomyelitis—bone infection; sinusitis—mucous membranes inflammation; peritonitis—inflammation of stomach inner wall; onychomycosis—shingles; etc.), *Penicillinium notatum* (a genus of fungi whose representatives are found in soil, on plants in the air, indoors, in the seas), and *Nematospora coryli* (a genus of fungi that causes sigmatonicosis—a disease affecting cotton, soybeans, pecans, pomegranates, citrus, and pistachio families).

The authors provide data on the inhibitory zone diameter of the compounds on a paper disk of 6 cm, inoculated (modified) by bacteria or fungi diffusion in agar of 50 micrograms per disk. Among all the tested structures, **DMHG**-based thiobazidalin analog **46c** has all types of fungi [54] and the greatest antimicrobial activity against Bacillus brevis (see Tables S2 and S3 in the Supplementary Materials).

**Figure 8:**
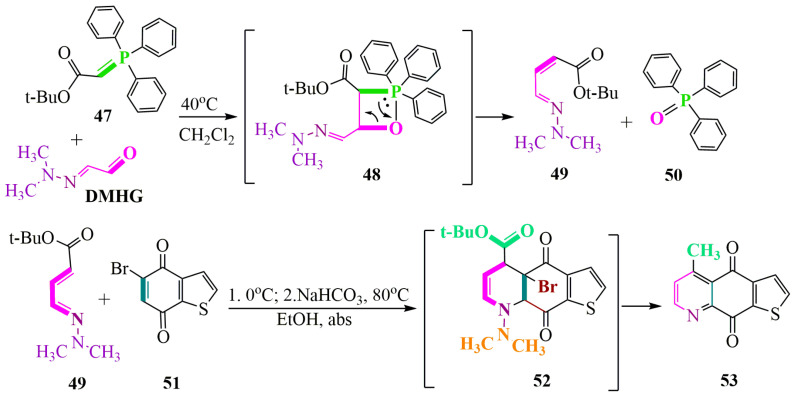
Antifungal methylthienoquinoline-4,9-dione synthesis.

8-Methylthieno[2,3-g]quinoline-4,9-dione, possessing antifungal activity, was synthesized from **DMHG** [55]. The synthesis involved two stages: the first stage comprised the Wittig reaction, followed by the second stage involving the Diels–Alder reaction (Figure 8). The Wittig reaction (Figure 8) was conducted in dichloromethane at a temperature of 40 °C. The desired product was obtained with a yield of 85.2% and subsequently purified using column chromatography with a diethyl ether:ethyl acetate mixture (5:1) as the eluent.

Tert-butylate **49** was also reacted with bromobenzothiophenedione **51** (Figure 8) at 0 °C to prevent its decomposition [56]. The synthesis was conducted in anhydrous ethanol solvent, in the presence of sodium carbonate, to eliminate hydrogen bromide and dimethylamine and to reduce the carboxyl group of intermediate **52**. This yielded the desired product, 5-methylthieno[3,2-g]quinoline-4,9-dione **53** (Figure 8).

**Figure 9:**
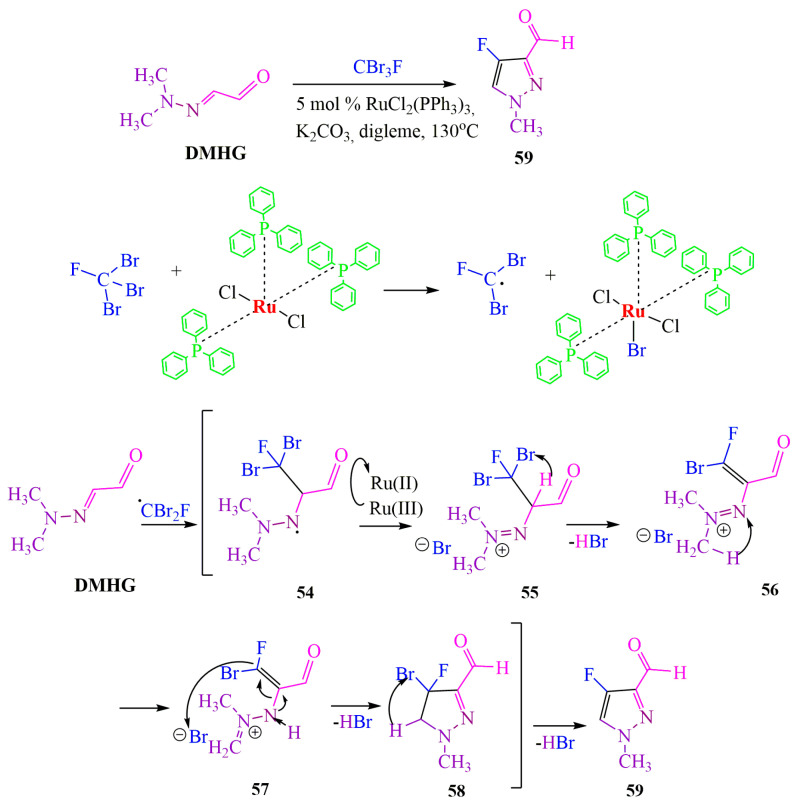
Redox mechanism of ruthenium catalyst.

**Figure 10:**
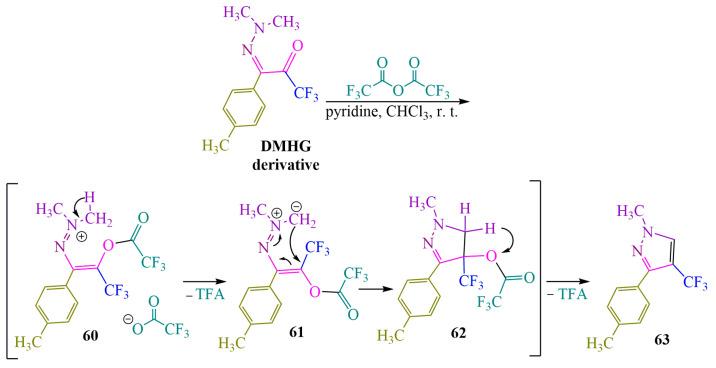
Cyclization of **DMHG** derivative into pyrazole.

**DMHG** [57] and its derivatives [58], which are applicable in the synthesis of fluorinated pyrazoles, are of interest in the pharmaceutical and agrochemical industries [59]. In the first case [57], the heterocycle was synthesized using ruthenium catalysis on a tribromofluoromethane basis (Figure 9), while in the second case [58], it was synthesized from trifluoroacetic anhydride (Figure 10).

The discussed derivatives can contribute to the trend in modern organic chemistry–cross-coupling reactions. Thus, in 2017 [57], a ruthenium-catalyzed synthesis of fluorinated pyrazole was proposed, as described in Figure 9. The authors suggested that the first stage of the chemical process involves the capture of the Ru(II) halide ion from CBr3F, resulting in the formation of a Ru(III) complex and a halide radical, CBr2F. The latter then interacts with **DMHG**, forming an aminyl radical **54**. Subsequently, Ru(III) transfers the halide ion to intermediate **54**, reducing to Ru(II) and forming diazene **55**. The basicity of diazene **55** catalyzes the elimination of hydrobromic acid, leading to the formation of compound **56**. Compound **56** then enolizes into imine ion **57**, which undergoes cyclization to form pyrazoline **58**. Finally, subsequent elimination of hydrogen bromide yields the desired product **59** (Figure 9).

The authors of publication [58] synthesized a fluorinated pyrazole through the reaction between a trifluoromethyl-containing **DMHG** derivative and trifluoroacetic anhydride in the presence of pyridine in chloroform at room temperature (Figure 10). The proposed cyclization pathway to form pyrazole **63** is as follows: Initially, trifluoroacetyl was attached to the carbonyl oxygen, resulting in the formation of salt **60**. Subsequently, methylide **61** underwent cyclization to form hydropyrazole **62**. Finally, the elimination of trifluoroacetic acid (TFA) led to the desired product **63** (Figure 10).

**DMHG**, upon reaction with hippuric acid **64**, undergoes a transformation leading to the formation of an isoxazole ring **66** [60]. In accordance with Lipinski’s rules [61], it exhibits similarities to pharmaceutical compounds. Its logP (logarithm of partition coefficient) value is 1.996, indicating unhindered penetration of isoxazole through both aqueous and lipid barriers toward the biological target.

**Figure 11:**
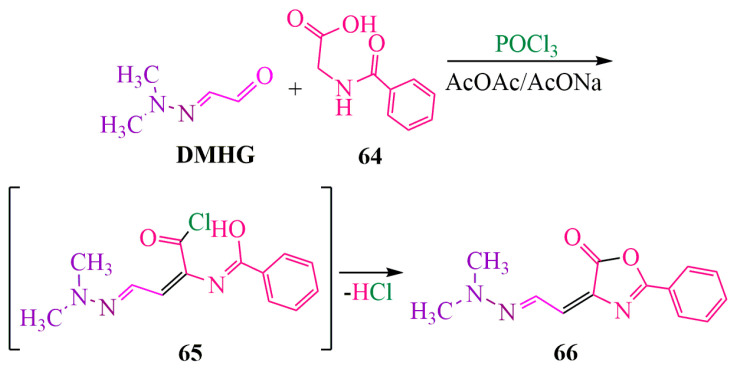
Cyclization of a hippuric acid derivative into isoxazole.

The synthesis of isoxazole [60] (Figure 11) was carried out in the presence of the chlorinating agent POCl3 in a mixture of acetic anhydride and a sodium acetate solution. **DMHG** was condensed with hippuric acid **64**, resulting in the formation of chlorohydrin **65**, which ultimately led to the desired isoxazole compound **66** (Figure 11).

**Figure 12:**
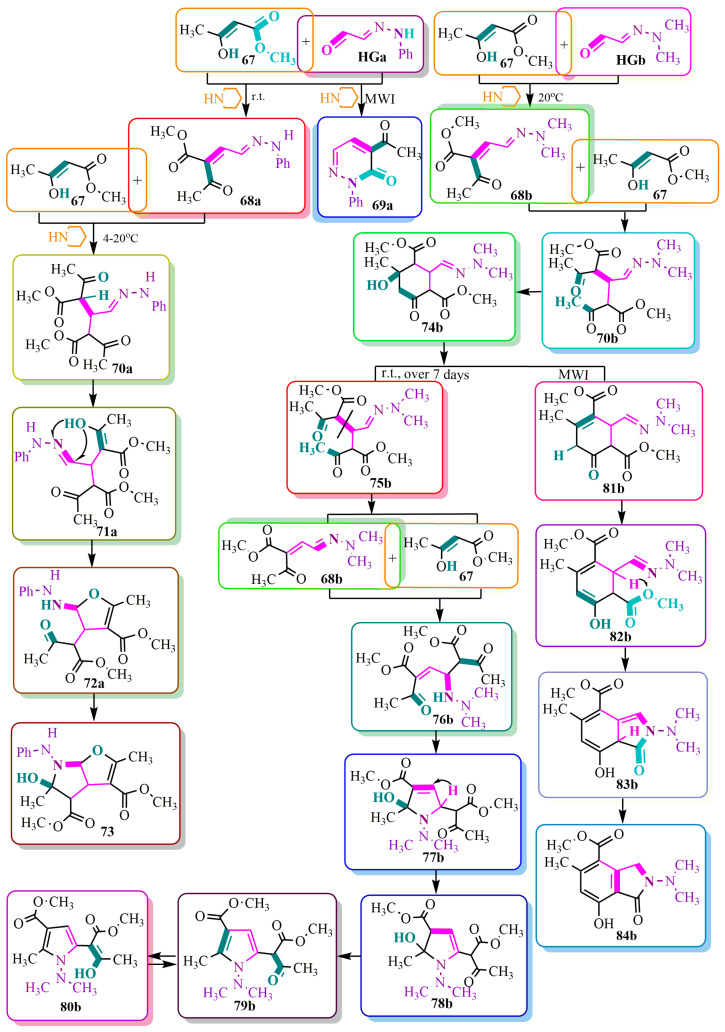
Piperidine-catalyzed syntheses under different conditions.

Interesting chemical transformations (Figure 12) are presented in publication [62], which explores solvent-free and pipyridine-catalyzed reactions under microwave irradiation (**MWI**), an effective method for dry organic syntheses [63,64,65,66,67,68,69,70,71,72]. Phenylhydrazone (**HGa**) and dimethylhydrazone (**HGb**) react with acetoacetic ether in a 1:1 ratio to form conjugated compounds **68a** and **68b**. Furthermore, the phenyl hydrazone derivative **68a** undergoes cyclization via methyl alcohol elimination under MWI catalysis, resulting in the formation of N-phenylpyridazine **69a**.

The reactions of condensation products **68a** and **68b** with acetoacetic ether **67** proceed differently. In the initial stage, both compounds share a common step where the enol form **67** adds to the double C-C bond of compounds **68a** and **68b**.

Subsequently, dimethylhydrazone **70b** forms an intermediate cyclohexanone **74b**, while the phenylhydrazone derivative **70a** undergoes an articulated furopyrrol **73** formation through the Robinson reaction [73].

The authors of [62] discovered that the cyclohexanone derivative **74b**, when left at room temperature for 7 days, underwent conversion into N-dimethylaminopyrrole **80b**. In the initial step, compound **74b** likely decomposes into the starting reagents **67** and **68b** through the opening of the cyclohexanone ring, followed by cleavage of the hydrazone bond **75b**. Subsequently, acetoacetic ether **67** adds to the C-N bond of compound **68b**, resulting in the formation of ether-ketone **76b**. The latter undergoes cyclization to form dihydropyrrole **77b**, while structure **78b** undergoes 1,3-hydride transfer followed by water elimination. This leads to the formation of pyrrole **79b** and enol compound **80b**, which exist in a tautomeric equilibrium.

However, in a counter-synthesis approach, microwave radiation of the original compound **74b** under conditions of 300 W and 160 °C for 30 min (equivalent to 7 days at room temperature) resulted in different outcomes. Instead of the expected product **80b**, a completely new benzopyrrole **84b** was formed. The transformations involved the elimination of methanol from cyclohexanone **74b**, followed by enolization of ketone **81b** and carbinol elimination from methyl ester **82b**. Eventually, a 1-3-hydride transfer in lactone **83b** led to the formation of an aromatic articulated heterocycle **84b** (Figure 12).

## 3. Dimethylhydrazones of Acrolein and Crotonal

Methylenedimethylhydrazones of acrolein **DMHA** (a colorless oil [74]) and crotonal **DMHC** (a colorless oil, 55–58 °C/15 Torr [75]) are building blocks for various nitrogen- and oxygen-containing heterocycles. **DMHA** and **DMHC** chemistry (namely, electron saturation [75]) allows them to be widely used in cycloaddition reactions [75,76,77].

### 3.1. DMHA and DMHC in Cycloaddition Reactions

**DMHA** was utilized in the synthesis of dihydro- [75] and tetrahydropyrane structures [78], which constitute components of diverse natural products [75,79]. These include cyclic saccharides obtained from coconut [79], irciniastatins (cytotoxins that induce necrosis within malignant neoplasm cells) isolated from sea sponges, exhibiting potential as anticancer agents [80], as well as a variety of marine products possessing a broad spectrum of biological activities such as antitumor, immunostimulatory, and analgesic properties [81].

In the contemporary scientific literature, the hydrazone methylene derivative (**DMHA**) has gained significant prominence as a fundamental component for constructing heterocyclic structures. In publication [75], this reagent was employed as a dienophile in the Diels–Alder reaction. The classical version of this reaction presents two primary challenges concerning α,β-unsaturated carbonyl compounds: (1) a substantial energy barrier between the diene and dienophile; (2) a lack of regioselectivity in the chemical process.

The former is explained by the energy sublevel discrepancy at the boundaries of molecular orbitals, which complicates the reaction between the reagents. The latter is caused by the fact that the highest occupied molecular orbital (HOMO) is occupied by electrons at the α and β positions. The electron arrangement facilitates cycloaddition simultaneously in two directions, leading to by-products.

**Figure 13:**
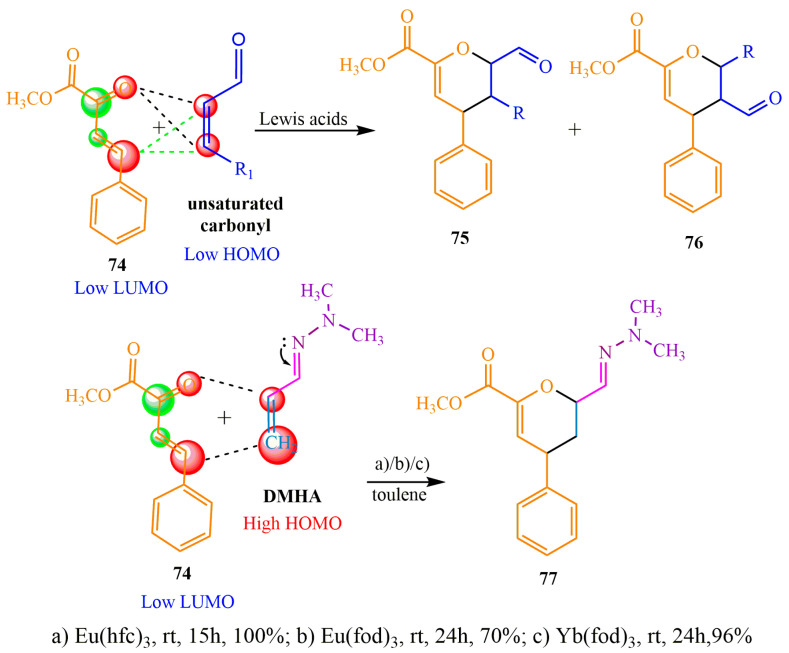
Classical Diels–Alder reaction in comparison with **DMHA**-based synthesis.

The authors of [75] proposed an enhancement to the Diels–Alder reaction technique by employing dimethylhydrazone of acrolein (**DMHA**) (Figure 13) as a dienophile. The **DMHA** imine’s electron-donating effect contributes to system saturation, thereby reducing the energy barrier between the reagents. To activate diene **74**, rare-earth metal salts were utilized as catalysts. The most favorable outcome was observed with heptafluorobutanol europium campherate (condition a), which is a widely employed catalyst in enantioselective Diels–Alder reactions. The reaction was carried out in toluene at room temperature for 15 h, resulting in the target product **77** with a quantitative yield (Figure 13).

**Figure 14:**
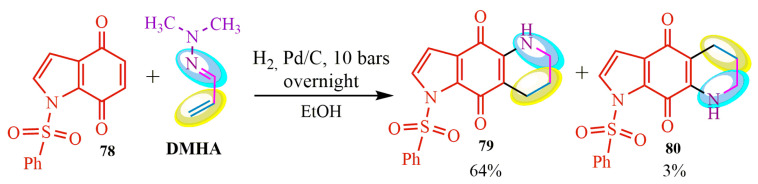
Diels–Alder reaction followed by hydrogenation.

A method has been developed for the one-step synthesis of piperidino-indoloquinolines, which are challenging to access. These compounds are key components of marine alkaloid discorhabdin C analogs, specifically hydrogenated Diels–Alder adducts **79** and **80** [78] (Figure 14). In this method, the DMHA-based Diels–Alder reaction with indoloquinone **78** was accompanied by a simultaneous selective reduction catalyzed by palladium under a hydrogen atmosphere in an alcoholic solution at a pressure of **10** bar overnight. The resulting reaction mixture was purified using chromatography with aluminum oxide as the sorbent. The desired products of this reaction were obtained as blue crystals, with yields of 64% for compound **79** and 3% for compound **80** (Figure 14).

**Figure 15:**
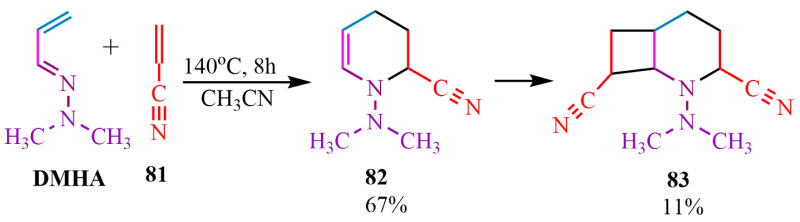
Bicyclic structure of diene synthesis.

The Diels–Alder reaction with DMHA as a diene was previously investigated ([82], 1992). It has been observed that acrylonitrile 81 participates in the DMHA-based diene synthesis not only through [4+2] cycloaddition but also through [2+2] cycloaddition. Consequently, bicyclooctane **83** was obtained in acetonitrile at a temperature of 140 °C with a yield of 11%. A similar reaction was conducted in benzene in the presence of hydroquinone at 120 °C, resulting in the formation of a six-membered product of diene synthesis **82** with a yield of 67% (Figure 15).

**Figure 16:**
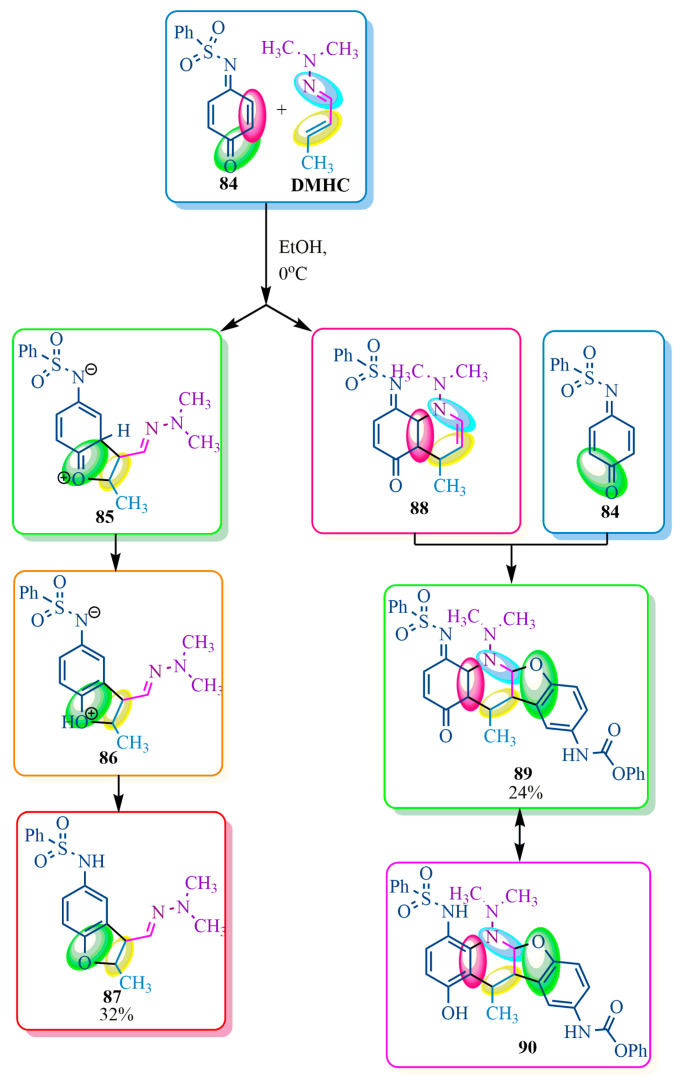
New direction of cycloaddition reaction.

However, the diene and dienophile cycloaddition reactions may exhibit different reaction pathways. Therefore, the authors of publication [76] investigated the interaction between quinonmonoimide **84** and dimethylhydrazadiene **DMHC** in ethanol at 0 °C. It was discovered that the reaction proceeded in two directions (Figure 16). One direction involved a [2+3]-cycloaddition, resulting in the formation of adduct **87**. The other direction involved a [2+4]-cycloaddition, yielding compound **88**. Upon reacting with a second molecule of quinon-imine **84**, the Diels–Alder adduct **88** produced a tetracyclic product **89**. The latter underwent slow isomerization to form aromatic aminophenol **90** in a deuterated chloroform solvent and was even slower in polar solvents such as acetone and ethyl acetate [76] (Figure 16).

### 3.2. DMHA and DMHC in Multicomponent Synthesis of Marine Alkaloids

**DMHA**-based literature describes various methods for obtaining pyridine structures that are marine alkaloids and their structural analogs with antitumor activity [77,78,83,84,85]. These include ascididemine [77,83] and tetrahydroascidemine [84].

**Figure 17:**
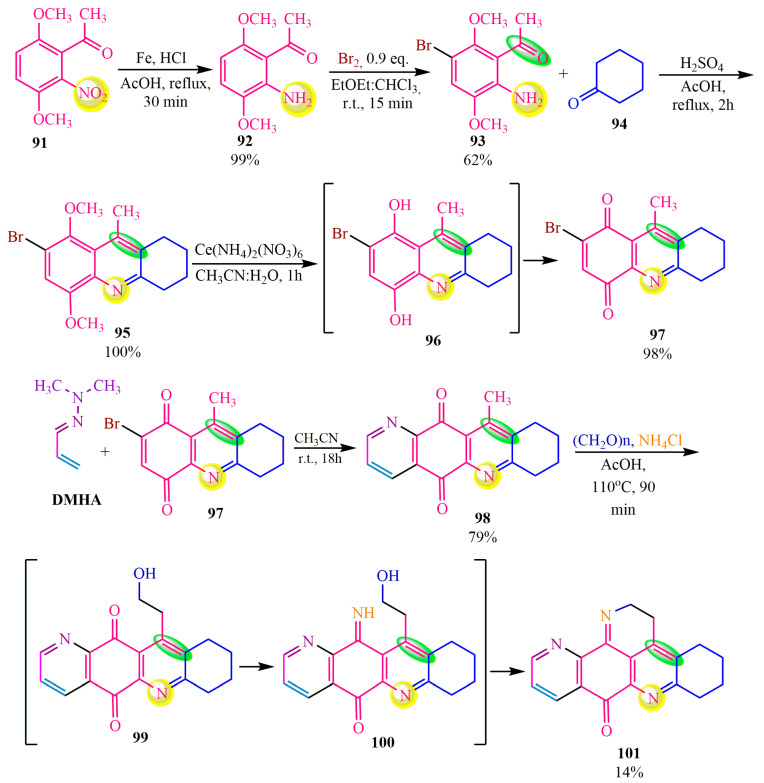
Synthesis of tetrahydroascidemine.

The synthesis of the latter compound **102** is described in publication [84] (Figure 17). The authors of [84] achieved the synthesis of compound **102** by reducing the nitro group of the initial ketone **91** to the corresponding amine **92** with a yield of 99% in the presence of iron in acetic acid and catalytic amounts of hydrogen chloride. Subsequently, amine **92** underwent halogenation in a mixture of ethyl ether and chloroform with a slight excess of bromine (1:0.9), resulting in the formation of target compound **93** with a yield of 62%. Brominated adduct **93** was then subjected to Friedlander’s reaction [86] with cyclohexanone **94**, leading to the formation of tricyclic product **95** with a yield of 100%. Compound **95** was further oxidized to quinone **97** using cerium ammonium nitrate (**CAN**) due to its ability to selectively affect ether functional groups, specifically the methoxy group, in this case [87]. The oxidation reaction was carried out in an aqueous acetonitrile medium, and compound **97** was obtained with a yield of 98%. Dienophile **97** was also employed in a hetero-Diels–Alder reaction with **DMHA**, yielding adduct **98** with a yield of 79%. Adduct **98** served as a methylene-active linker in the subsequent Mannich reaction, leading to the formation of the target compound **101** with a yield of 14% (Figure 17).

The article [84] presents data on the antitumor activity of synthesized derivatives **98** and **101** in comparison to the known alkaloid ascididemine [84] against four cell lines (Table S5). The compounds exhibited an inhibitory concentration of 50% in cell lines at approximately 10−6 M [84] (see Table S4 in the Supplementary Materials).

**Figure 18:**
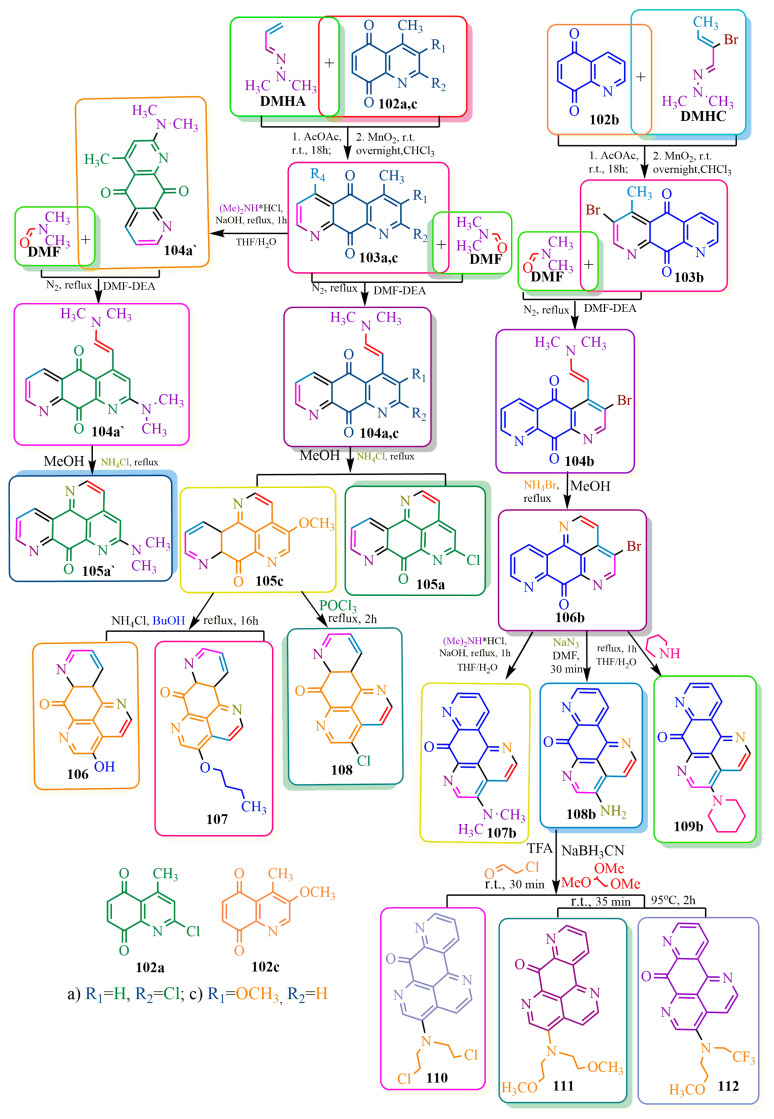
**DMHA**- and **DMHC**-based synthesis of phenantroline-7-one derivatives.

Moreover, Ascididemine derivatives with enhanced efficacy against oncology were synthesized [85] (Figure 18). In the initial step, a [4+2]-cycloaddition reaction was performed using dienes **DMHA** and **DMHC** as well as dienophiles **102a**, **102b**, and **102c**. Subsequent elimination reactions via acetic anhydrid and manganese dioxide [88] yielded adducts **103a**, **103b**, and **103c**. Among these, adduct **103a** was specifically chosen for the synthesis of its dimethylamino derivative **104a’**. This was achieved by employing dimethylamine in hydrochloride form (due to the gaseous nature of the amine), followed by an alkali treatment to neutralize the reaction medium. A solvent mixture of water and tetrahydrofuran was utilized, where water facilitated the dissolution of hydrochloride and alkali, while nonpolar tetrahydrofuran prevented undesired side reactions such as pyridinium salt formation. Compounds **104a’**, **103a**, **103b**, and **103c** were subsequently employed in further chemical transformations [85]. The subsequent stage involved elements of the Bracher method [85,89]. A combination of polar and basic solvents, namely dimethylformamide (**DMF**) and diethanolamine (**DEA**), was used to promote the condensation of compounds **103a**, **103b**, **103c**, and **104a’** with **DMF** under an inert nitrogen atmosphere. This led to the formation of intermediates **104a**, **104b**, **104c**, and **105a’**. These intermediates then underwent cyclization to yield the desired products **105a**, **105c**, **106a’**, and 1**06b** (Figure 18). The bromine atom of phenanthroline-7-one derivative **106b** [85] was substituted with various amino groups (Figure 18). Dimethylamino and N-piperidino groups were introduced in an aqueous THF solution under basic conditions, resulting in the formation of compounds **107b** and **109b**, respectively. Amine **108b** was obtained from sodium azide. Subsequent chemical transformations of compound **108b** were carried out using aldehydes and acetals in the presence of sodium boron anhydride and **TFA**, yielding compounds **110**, **111**, and **112**. Chlorine (compound **108**) was incorporated into structure **105c** through the use of phosphoric acid chlorohydride. Hydroxyl and butyl groups (structures **106** and **108**, respectively) were introduced by reacting with butyl alcohol in the presence of ammonium chloride [85] (Figure 18).

The synthesized marine alkaloid analogs were tested on 12 cancer cell lines [87]. The IC_50_ value of these compounds on 12 cell lines made 10^−6^ M. (see Table S5 in the Supplementary Materials).

### 3.3. Unusual DMHA Reaction (Elongation of the Hydrocarbon Chain)

**Figure 19:**
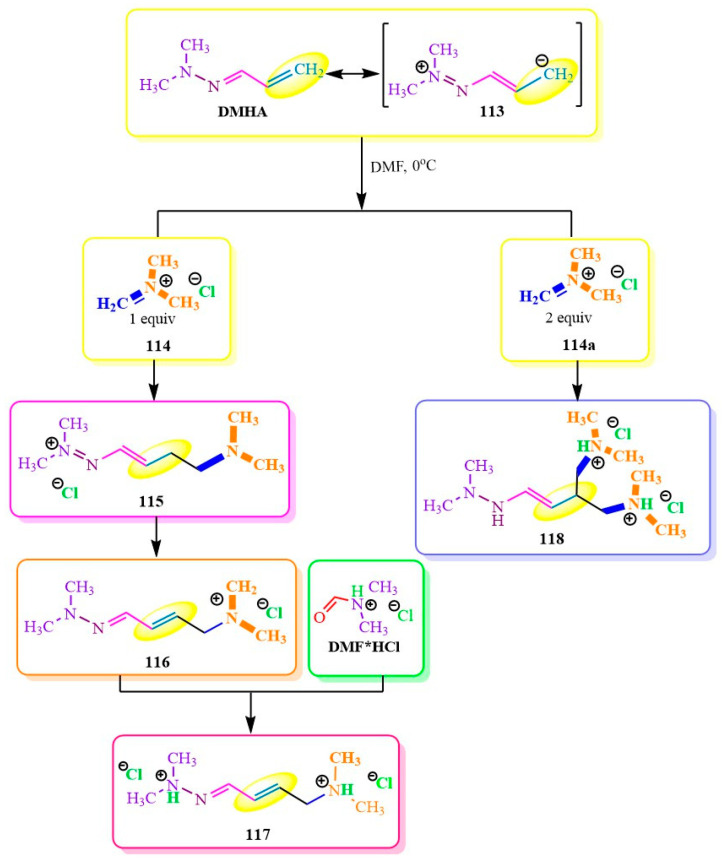
Amination of N,N-dimethylaminforminium chloride.

The **DMHA** chain lengthening described in publication [90] (Figure 19) is also of interest in organic synthesis. *N,N*-dimethylformiminium hydrochloride **114** in absolute dimethylformamide (**DMF**) was used as the electrophile. The authors of [90] proposed that **DMHA** adds to *N,N*-dimethylformiminium **114** (Figure 19) through its tautomeric form **113**, with the hydrogen being replaced by the methylene group of intermediate **115** located at the dimethylamino group. When one equivalent of N,N-dimethylformiminium **116** was added to **DMHA**, salt **117** was formed. Crystallization of salt **117** was achieved by adding DMF*HCl, resulting in the formation of dihydrochloride product **117**. However, when twice the amount of the same reagent **114a** was added, compound **118** crystallized independently without salting out (Figure 19).

## 4. Methacrolein Dimethylhydrazone

Dimethylhydrazone methacrolein (**DMHM**) (colorless oil, b.p. 40–42 °C/20 Torr [91]) is also applicable in the synthesis of the tetrahydroquinoline ring [92,93], which is a part of various natural products. These include benzostatins that prevent lipid peroxidation, thereby reducing the likelihood of patagenesis [94] and reducing the toxicity of glutamate [95], of cusparin and allocusparin having anti–tuberculosis activity [96], and martellinic acid activity against conjunctivitis [97].

**Figure 20:**
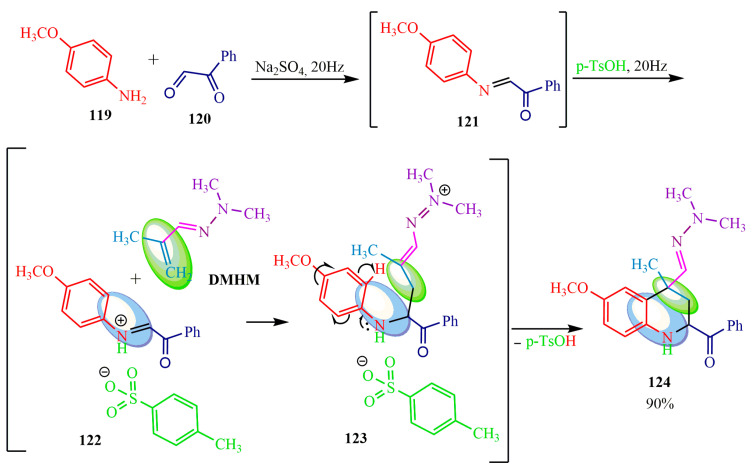
Modified Povarov reaction.

In 2021, the authors of publication [92] reported the potential enhancement of benzostatin derivatives **119** yield (90%) through the utilization of a 20 Hz vibrating ball mill and appropriate catalyst selection in the Povarov reaction (Figure 20). Initially, Schiff base **121** was synthesized by reacting p-anisidine **119** with phenylglyoxal **120**. Subsequently, the reaction with methylacrolein **DMHM** was catalyzed by tosylic acid (p-TsOH). The catalytic process likely proceeded as follows: firstly, the proton p-TsOH was localized at the imine **121**, resulting in the formation of tosylate **122**. Secondly, the addition of **DMHM** led to electron density and proton migration from the phenyl ring **123**. Consequently, tosylic acid was regenerated, followed by the cyclization of aryl **123** to tetrahydroquinoline **124** (Figure 20).

**Figure 21:**
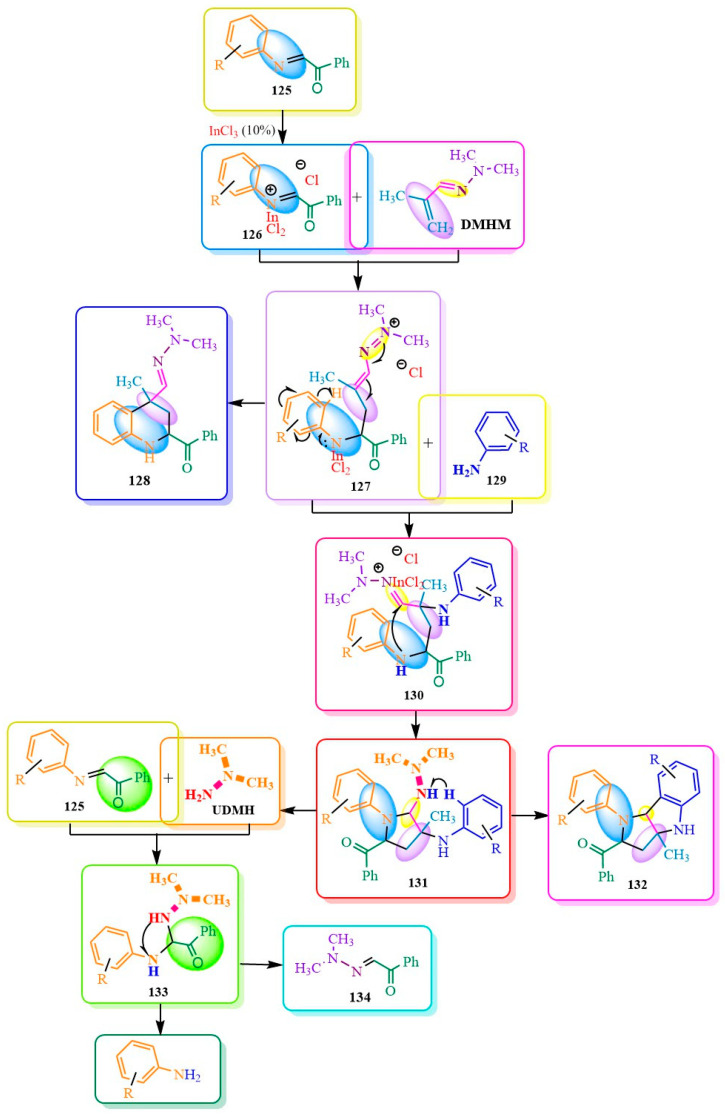
Directions of reaction according to Povarov type.

The synthetic capabilities of **DMHM** (dimethylhydrazone methacrolein) in the Povarov reaction were previously described in 2012. In their publication [93], the authors investigated the pathways for two-component and three-component syntheses (Figure 21). In the first case, tetrahydroquinoline **128** was synthesized through the indium (III) chloride-catalyzed reaction of **DMHM** with Schiff base **125** in acetonitrile at room temperature. In the second case, the authors of [90] elucidated the formation of a tricyclic structure **132** from arylamine **135**. This was explained by a cascade process involving several steps. Firstly, compound **129** was added to the double C-C and C-N bonds of compound **127**. Subsequently, an intramolecular cyclization of the amino group occurred via the double bond of hydrazone **130**, leading to simultaneous catalyst regeneration. Following this, product **131** underwent cyclization with the elimination of asymmetric dimethylhydrazine (**UDMH**). The **UDMH** then underwent transamination with the original compound **133**, resulting in the formation of dimethylhydrazon **134** (Figure 21).

**Figure 22:**
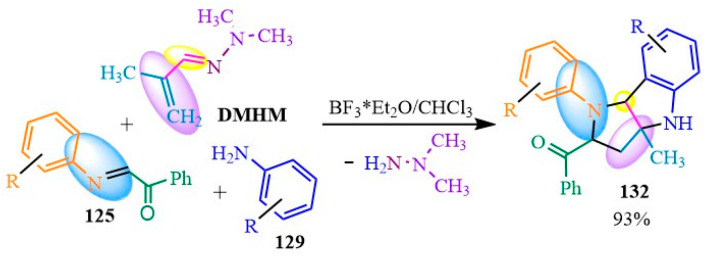
Tricyclic derivative **132** yields Amination of N,N-dimethylaminforminium chloride.

To enhance the yield of the tricyclic derivative **132**, the researchers of [93] utilized the BF3*Et2O/CHCl3 catalyst/solvent system, resulting in a 93% yield of the desired compound. In certain instances, minor quantities of transamination products and tetrahydroquinoline were also obtained (Figure 22). Conducting the synthesis in a concentrated solution of the same system, as anticipated, increased the yield of condensate **132** but led to the formation of diastereomers. The excess arylamine contributed to transamination reactions rather than cyclization towards the desired compound **132 [93]** (Figure 22).

The structure **132** bears a resemblance to ethyl 7-fluoro-3,4-dihydropyrrolo[3,4-b]indoles, which exhibit neuroleptic activity [98], and 3,4-dihydropyrrolo[3,4-b]indol-1(2H)-ones, known as serotonin receptor agonists [99,100]. **DMHM** finds utility in the synthesis of anthracycline structures, which hold significant significance in oncology treatment [101].

## 5. Formaldehyde Dimethylhydrazone

Formaldehyde Dimethylhydrazone (**MDH**) is also of interest in creating valuable organic compounds. Thus, on this basis, a beta-lactam scaffold obtained [102] is widely used in medicine as an antibacterial agent [103] and as an inhibitor of serine protease [104], human leukocyte elastase [105], cytomegalovirus protease [106], thrombin [107], prostate-specific antigen [108], cholesterol absorption [109], and tryptases [110]. Some β-lactams also showed antitumor activity [111].

**Figure 23:**
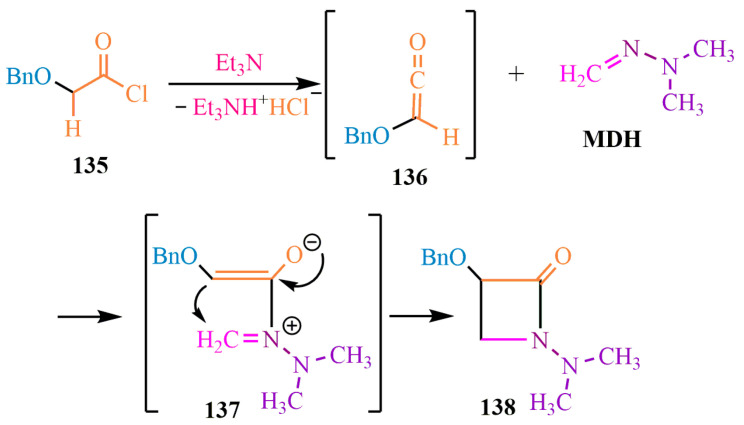
Four-membered heterocycle formation.

The synthesis of the hard-to-reach azetidine cycle [101] (Figure 23) was accomplished via hydrochloric acid elimination, followed by **MDH** addition to the ketene **136** C=O bond. The electron density in intermediate **137** facilitated intramolecular cyclization to form azetidine **138** (Figure 23).

**Figure 24:**
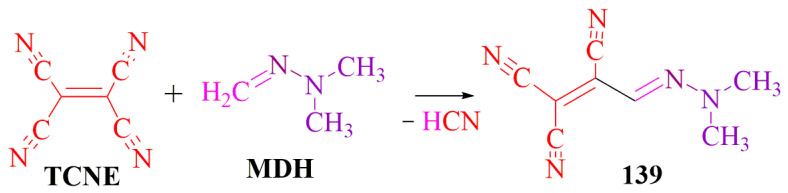
TCNE and MDH interaction.

**TCNE** (tetracyanoethylene) undergoes a reaction with the mobile hydrogen of the **MDH** (methylene active link) moiety, as depicted in Figure 24. Simultaneously, tricyanohydrazone derivatives are formed, which are recognized as promising antimicrobial dyes and photosensitizers [15] (Figure 24).

**Figure 25:**
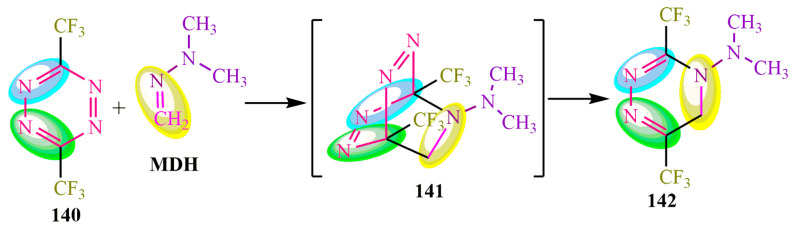
Cycloaddition [4+2].

**MDH** is capable of undergoing [4+2]-cycloaddition reactions, specifically Alder–Rickett-type reactions (Figure 25), with a tetrazene derivative **140**. This reaction leads to the formation of a bicyclic structure **141**, which can be cleaved to release nitrogen and subsequently form triazinamine **142** [112] (Figure 25).

## 6. Conclusions

Thus, hydrazones of dimethylhydrazine carbonyl derivatives hold promise in the creation of various natural structure analogs (alkaloids, enzymes, antibacterial, antitumor drugs), as well as serving as a tool for molecular design in organic synthesis.

The treatment of substandard rocket fuel through the formation of hydrazones of carbonyl compounds offers several advantages:

1. Negative cost of the original unsymmetrical dimethylhydrazine (UDMH);
2. Reduced toxicity and less pungent odor of carbonyl derivatives (compared to UDMH itself), facilitating their use in large-scale and multi-stage synthesis;
3. The possibility of conducting stereoselective reactions and obtaining optically pure compounds;
4. The electron-rich nitrogen–carbon double bond enables various cycloaddition reactions (4+2, 3+2, 2+2) and the synthesis of heterocyclic derivatives with high yields.
5. Many heterocyclic compounds based on dimethylhydrazone have demonstrated high antitumor activity (phenanthroline-7-ones), antifungal activity, and antibacterial activity (thiobazidalin derivatives);
6. In numerous reactions, target products with quantitative yields have been obtained. For example, dihydropyran, a component of irciniastatins (marine products), can be synthesized via the Diels–Alder reaction with a 100% yield (Eu(hfc)3, room temperature, 15 h). The Povarov reaction can provide a tetrahydroquinoline ring, which is a constituent of benzostatins. However, one of the main drawbacks of using DMH carbonyl derivatives in organic synthesis is the requirement for hard-to-access reagents (LDA, Bu-Li, t-BuOK, InCl2, AcOAc, Eu(hfc)3) for the transformation into target compounds.

## Figures and Tables

**Table 1:** Reagents and conditions for synthesis of compounds **45a,b,c.**

Reagents	Conditions	Products
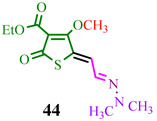	**Dry gaseous NH_3_, −10 °C, EtOAC:EtOH (1:1)**	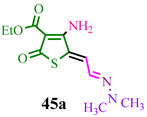
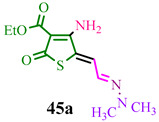	**Dry acetonitrile, N_2_, reflux, 1 h**	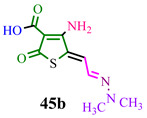
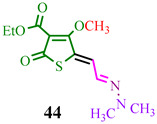	**CH_3_NH_2_, −10 °C, EtOAC:EtOH (1:1)**	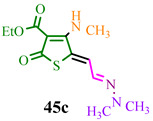

## Data Availability

Data are contained within the article and Supplementary Materials.

## Supplementary Materials

The following supporting information can be downloaded at https://www.mdpi.com/article/10.3390/ijms242417196/s1.
