# Supplementary material for: The Recycling of Substandard Rocket Fuel N,N-Dimethylhydrazine via the Involvement of Its Hydrazones Derived from Glyoxal, Acrolein, Metacrolein, Crotonaldehyde, and Formaldehyde in Organic Synthesis
Source: Int J Mol Sci. 2023 Dec 6;24(24):17196. doi: 10.3390/ijms242417196 (PMC10742919; doi:10.3390/ijms242417196)
Supplement: Supplementary file 1 [file ijms-24-17196-s001.zip › ijms-2689058-supplementary.pdf]

Supporting information

Recycling of Substandard Rocket Fuel N,N-Dimethylhydrazine via the Involvement of Its Hydrazones Derived from Glyoxal, Acrolein, Metacrolein, Crotonaldehyde, and Formaldehyde in Organic Synthesis.

Elizaveta Ivanova [1], Margarita Osipova [1], Tatyana Vasilieva [1], Alexey Eremkin [1], Svetlana Markova [1], Ekaterina Zazhivihina [1], Svetlana Smirnova [1], Elena Turusova [1], Yurii Mitrasov [2] and Oleg Nasakin [1,]*

[1] *Ulyanov Chuvash State University, Moskovsky pr., 15, Cheboksary 428015, Russia*

[2] *Organic and Pharmaceutical Chemistry Department, Yakovlev Chuvash State Pedagogical University, K. Marx Street, 38, 428000, Cheboksary, Russia*

**Corresponding author. Tel.: +7 903 345 57 33 (Oleg E. Nasakin); +7 961 340 01 81 (Elizaveta S.Ivanova).*

E-mail address: ecopan21@inbox.ru (O.E. Nasakin), lizachimic@mail.ru (E.S. Ivanova)

Table S1. Inhibitory activity against kinaza p38α, IC50*10^{-6}M.

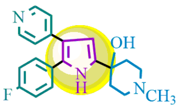	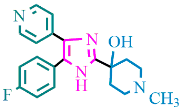	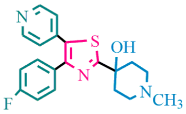	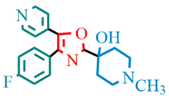	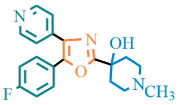
**0.13**	**0.09**	**0.45**	**0.35**	**2**

Table S2. Antibacterial activity. Diameter of the inhibitory zone (nm).

**Compounds**	***Mucor miehei***	***Paecilomyces varioti***	***Penicillium notatum***	***Nematospora coryli***
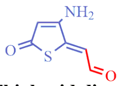 **Thiobasidalin**	**15**	**11**	**-**	**16**
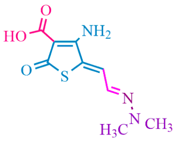	**-**	**-**	**-**	**-**
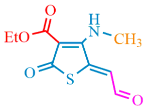	**20**	**20**	**12**	**22**
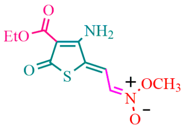	**15**	**11**	**-**	**13**
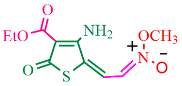	**18**	**15**	**-**	**15**
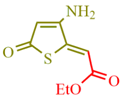	**11**	**10**	**-**	**9**
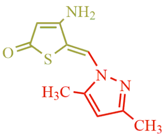	**9**	**9**	**-**	**10**

Table S3. Antifungal activity. Diameter of the inhibitory zone (nm).

Compounds	*Bacillus subtilis*	*Bacillus brevis*	*Micrococcus luteus*	*Nematospora coryli*
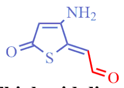 **Thiobasidalin**	-	-	-	-
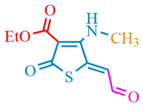	10	19	-	-
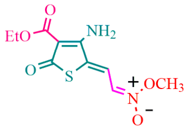	8	-	-	-
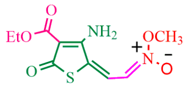	19	-	-	20

Table S4. Antitumor activity of compounds **98** and **101** (IC50*10^{-6}M).

Compounds	p-388	A-549	HT-29	MEL-28
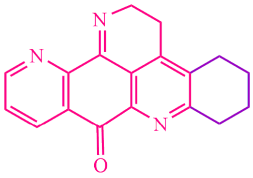	1.74	0.04	0.3	0.17
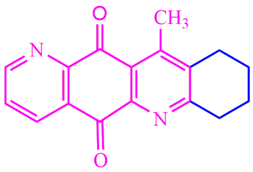	>35.9	45	18.0	18.0
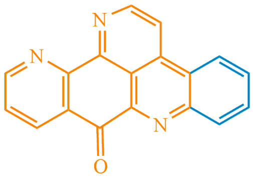 **Ascididemin**	0.4[81]		0.3[81]	

Table S5. Antitumor activity ($IC_{50}*10^{-6}M$).

Compounds	U-87MG	U-373MG	SW1088	T24	J82	HCT-15	LoVo	MCF7	T-47D	A-549	A-427	PC-3
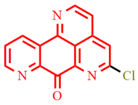	5	5	7	6	>10	6	6	7	>10	6	5	>10
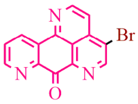	2	5	5	6	6	4	5	4	5	7	4	6
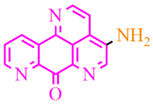	3	5	4	4	>10	6	6	2	9	5	7	7
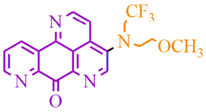	0.9	0.4	0.1	0.08	4	0.7	0.2	0.3	0.9	6	2	0.7
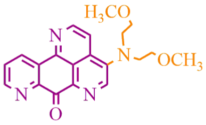	0.9	0.7	0.009	0.009	>10	0.9	0.8	0.5	>10	1	0.08	0.007
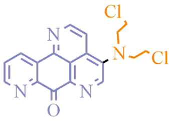	0.09	0.05	0.05	0.02	0.5	0.08	0.03	0.02	0.6	0.5	0.4	0.05
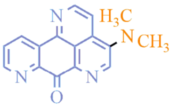	0.7	0.6	0.5	0.5	0.7	0.9	4	0.5	5	0.6	0.3	0.6
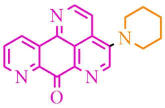	0.08	0.3	0.002	0.0004	5	0.09	0.06	0.009	0.006	0.7	0.006	0.0004
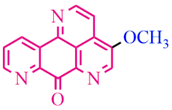	0.05	0.07	0.08	0.06	0.8	0.09	0.7	0.06	6	0.05	0.07	0.08
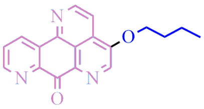	2	0.6	4	0.7	4	3	4	0.8	4	6	0.6	0.5
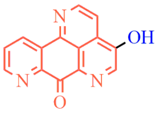	>10	>10	>10	>10	>10	>10	>10	>10	>10	8	>10	>10
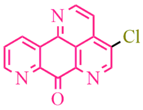	6	5	5	5	3	6	6	2	4	5	3	6
